# The association between acute fatty liver disease and nitric oxide during malaria in pregnancy

**DOI:** 10.1186/s12936-021-03999-2

**Published:** 2021-12-14

**Authors:** Mamoru Niikura, Toshiyuki Fukutomi, Shoichiro Mineo, Jiro Mitobe, Fumie Kobayashi

**Affiliations:** 1 Department of Infectious Diseases, Kyorin University School of Medicine, 6–20–2 Shinkawa, Mitaka, Tokyo, 181–8611 Japan; 2 Department of Pharmacology and Toxicology, Kyorin University School of Medicine, Tokyo, Japan; 3 Department of Molecular Pathology, Tokyo Medical University, Tokyo, 160–8402 Japan; 4 Department of Environmental Science, School of Life and Environmental Science, Azabu University, Kanagawa, 252-5201 Japan

**Keywords:** Fatty liver, iNOS, Liver disease, Malaria, Pregnancy, Proteome

## Abstract

**Background:**

Liver disease is a common feature of malaria in pregnancy, but its pathogenesis remains unclear.

**Methods:**

To understand the pathogenesis of liver disease during malaria in pregnancy, comparative proteomic analysis of the liver in a mouse model of malaria in pregnancy was performed.

**Results:**

Decreased levels of mitochondrial and peroxisomal proteins were observed in the livers of pregnant mice infected with the lethal rodent malaria parasite *Plasmodium berghei* strain NK65. By contrast, increased levels of perilipin-2, amyloid A-1, and interferon (IFN)-γ signalling pathway-related proteins were observed in the livers of infected pregnant mice, suggesting that IFN-γ signalling may contribute to the development of liver disease during malaria in pregnancy. IFN-γ signalling is a potential trigger of inducible nitric oxide synthase (iNOS) expression. Liver disease associated with microvesicular fatty infiltration and elevated liver enzymes in pregnant wild-type mice infected with malaria parasites was improved by iNOS deficiency.

**Conclusions:**

In this study, a causative role of iNOS in liver disease associated with microvesicular fatty infiltration during malaria in pregnancy was demonstrated. These findings provide important insight for understanding the role of iNOS-mediated metabolic responses and the pathogenesis of high-risk liver diseases in pregnancy, such as acute fatty liver.

**Supplementary Information:**

The online version contains supplementary material available at 10.1186/s12936-021-03999-2.

## Background

Individuals living in malaria-endemic regions acquire protective immunity against malaria parasites and often show asymptomatic infection. However, women are highly susceptible to malaria infection because of low immunity during pregnancy [1, 2]. Malaria during pregnancy, in addition to maternal illness or death, is implicated in placental malaria, which has been correlated with adverse pregnancy outcomes [3–5]. Malaria in pregnancy is a major public health problem in endemic areas, especially in Africa [6].

Severe anaemia, thrombocytopenia, and liver dysfunction are common features of severe malaria [7]. These pathological features of pregnant women infected with *Plasmodium falciparum* can be confused with haemolytic anaemia, elevated liver enzymes, and low platelet count (HELLP) syndrome [8–10]. Similar to HELLP syndrome, pregnant women infected with malaria parasites undergo spontaneous abortion, stillbirth, and premature delivery [3–5]. In North-western Colombia, hepatic dysfunction is the most frequent complication in patients with severe cases (9 of 15 patients) [11]. These findings indicate that hepatic dysfunction is common during human placental malaria.

In a study using a mouse model of malaria in pregnancy, when mice immunized with the attenuated malaria parasite *Plasmodium berghei* strain XAT were infected with the lethal malaria parasite *P. berghei* strain NK65, they showed severe liver disease and placental pathology in the late phase of pregnancy, resulting in adverse pregnancy outcomes [12]. Histological analysis revealed microvesicular fatty infiltration in livers obtained from the mouse model of malaria in pregnancy. Liver disease associated with the deposition of fat showed pathological features similar to those of acute fatty liver of pregnancy (AFLP).

Differentiation of HELLP syndrome and AFLP can be challenging, because the initial pathological features of these diseases are similar [13]. It is believed that defects in fatty acid metabolism during pregnancy are involved in acute fatty liver development [14]. Indeed, approximately 20% of cases of AFLP have been associated with long-chain 3-hydroxyacyl CoA dehydrogenase deficiency [15, 16]. However, to date, the detailed mechanisms by which pregnant women develop HELLP syndrome and AFLP are not fully understood.

The study of a mouse model exhibiting liver disease associated with microvesicular fatty infiltration during malaria in pregnancy may help improve our understanding of the pathogenesis of liver diseases in pregnancy, such as AFLP. To investigate the pathogenesis of acute fatty liver disease in pregnancy, a comparative proteomic analysis of the liver in a mouse model of malaria in pregnancy was performed. The effects of inducible nitric oxide synthase (iNOS) deficiency on acute fatty liver disease during malaria in pregnancy were also investigated using the mouse model.

## Methods

### Animals and ethics

Female and male C57BL/6 J (B6) mice (5–6 weeks of age) were purchased from CLEA Japan Inc. (Tokyo, Japan). iNOS-deficient mice [17] were a gift from Dr. Takuya Sakurai (Department of Molecular Predictive Medicine and Sport Science, Kyorin University, School of Medicine). The experiments were approved (#220) by the Experimental Animal Ethics Committee of Kyorin University School of Medicine. All experimental animals were maintained in the animal facility in a specific pathogen-free unit with sterile bedding, food, and water. A female mouse > 9–11 weeks of age was mated for 1 day with a male mouse in a cage and examined for the presence of a vaginal plug the next morning.

The infection studies included frequent observations to determine humane endpoints, such as an inability to ambulate sufficiently to obtain water or food. At the indicated time points, mice were euthanized by cervical dislocation under isoflurane or pentobarbital sodium anaesthesia. All experiments were designed to minimize animal suffering. When illness or death was expected due to experimental infections, mice were visually checked by investigators at least twice daily (including weekends and holidays). The investigators who conducted the experiments had completed the Experimental Animal Ethics Committee training course on animal care and handling. All experiments were performed in accordance with the guidelines and regulations of animal experimentation at Kyorin University. All animal experiments were also carried out in compliance with the ARRIVE guidelines.

### Parasites and infections

Lethal *P. berghei* NK65 and attenuated *P. berghei* XAT parasites were stored as frozen stocks in liquid nitrogen [12, 18]. Malaria parasite-infected erythrocytes were generated in donor mice inoculated intraperitoneally with a frozen stock of parasite. The donor mice were monitored for parasitaemia daily and bled for experimental infection during periods of ascending parasitaemia. Female mice 5–6 weeks of age were injected intravenously with 1 × 10^4^ erythrocytes infected with attenuated parasites. On day 29 post-infection, these female mice were mated with a > 9-week-old male mouse for 1 day and examined for the presence of a vaginal plug the following morning (immunized (IM) pregnant and non-pregnant mice). Mice immunized with attenuated parasites with or without a vaginal plug were infected intravenously with 1 × 10^4^ erythrocytes infected with lethal parasites (IM pregnant and non-pregnant mice infected with *P. berghei* NK65).

### Parasitaemia

Methanol-fixed tail blood smears stained with 3% Giemsa diluted with phosphate buffer (pH 7.2) for 45 min were observed under a microscope. The number of infected erythrocytes in 250 erythrocytes was counted when parasitaemia exceeded 10%, and 1 × 10^4^ erythrocytes were examined in mice with milder parasitaemia. The percentage of parasitaemia was calculated as follows: [(number of infected erythrocytes)/(total number of erythrocytes)] × 100.

### Histological examination and measurement of parameters of liver injury

Livers and blood were obtained from uninfected pregnant and non-pregnant mice, and IM pregnant and non-pregnant mice infected with *P. berghei* NK65, on day 17 post-mating. Note that “uninfected mice” were neither immunized with *P. berghei* XAT nor infected with *P. berghei* NK65. Placentas and fetuses were obtained from uninfected pregnant mice, IM pregnant mice, and IM pregnant mice infected with *P. berghei* NK65 on day 17 post-mating. Tissues and fetuses were fixed in 10% buffered formalin and embedded in paraffin, or fixed in 4% paraformaldehyde and frozen at − 80 °C. Sections (6 µM thick) were stained with haematoxylin and eosin or Sudan IV. The latter sections were photographed at 400 × magnification using an All-in-one Fluorescence Microscope (BZ-9000; KEYENCE Japan, Osaka, Japan). Fat droplets in the photographs were counted using BZ-II Analyzer software (KEYENCE Japan). Blood was centrifuged at 500×*g* for 10 min. The resulting supernatants were stored at − 20 °C and used as plasma. The levels of aspartic aminotransferase, alanine aminotransferase, and glucose in plasma were determined at Nagahama Life Science Laboratory (Shiga, Japan).

### Comparative proteomic analysis

Livers were obtained from uninfected pregnant and non-pregnant mice, and IM pregnant and non-pregnant mice infected with *P. berghei* NK65 on day 17 post-mating. Proteins were extracted using the Mammalian Protein Extraction Reagent (Thermo Fisher Scientific, Waltham, MA, USA) according to the manufacturer’s protocol and treated with trypsin. Comparative proteomic analyses were performed as previously described [19, 20]. The fractionated peptides were injected into a trap column (C18, 0.3 × 5 mm; L-column, Chemicals Evaluation and Research Institute, Tokyo, Japan) and an analytical column (C18, 0.075 × 120 mm; Nikkyo Technos, Tokyo, Japan) attached to a nano liquid chromatography-tandem mass spectrometry (nanoLC-MS/MS) system. The nanoLC-MS/MS analysis was conducted using an LTQ Orbitrap Velos mass spectrometer (Thermo Fisher Scientific) equipped with a nanoLC interface (KYA, Tokyo, Japan) and a nano high-performance liquid chromatography (nanoHPLC) system (DiNa; KYA). Purified peptides from the nanoLC were introduced into the LTQ Orbitrap Velos, a hybrid ion-trap Fourier transform mass spectrometer. Full MS and MS/MS scans were followed by higher-energy collisionally activated dissociation. The database search engines Proteome Discoverer 1.4 (Thermo Fisher Scientific) and MASCOT 2.6 (Matrix Science) were used to identify and quantify proteins from the MS, MS/MS, and reporter ion spectra of peptides.

Peptide mass data were matched by searching the NCBInr database. The false discovery rate (FDR) [21] was calculated via peptide sequence analysis using Percolator [22]. High-confidence peptide identifications were obtained by setting a target FDR threshold of ≤ 1.0% at the peptide level. Proteins showing one or two peptide spectral matches (PSMs) were excluded. Protein levels were normalized to actin, cytoplasmic 1 (accession: P60710), as previously described [23]. The normalized experimental signal was calculated as follows: (observed experimental signal) ÷ (normalisation factor).

### Enzyme-linked immunosorbent assay (ELISA) of cytokines

An ELISA for the detection of IFN-γ or IL-10 in plasma was carried out as described previously [18]. A rat anti-mouse IFN-γ mAb (clone R4–6A2; eBioscience, San Diego, CA, USA) and a rat anti-mouse IL-10 (clone JES5–16E3; eBioscience) were used as the capture antibodies, and a biotinylated, rat anti-mouse IFN-γ mAb (clone XMG1.2; eBioscience) and rat anti-mouse IL-10 mAb (clone JES5–2A5; eBioscience) as the detecting antibodies. The concentrations of cytokines in plasma were calculated from standard curves prepared using known quantities of murine recombinant IFN-γ (Genzyme, Boston, MA, USA) and IL-10 (Pierce, Rockford, IL, USA). The reaction was visualized using peroxidase-conjugated streptavidin (Zymed) and the substrate 2,2’-azino-bis(3-etylbenzthiazoline-6-sulphonic acid) (Wako, Osaka, Japan).

### Statistical analysis

For time-series comparisons, Student’s *t*-test and one- and two-way analyses of variance with Fisher’s protected least significant difference post-hoc test (Tukey–Kramer and Dunnett test) were performed using the Statcel program (OMS, Saitama, Japan). *P*-values < 0.05 were considered statistically significant.

## Results

### Proteomic analysis of the liver of immunized pregnant mice infected with *P. berghei* NK65

**Fig. 1:**
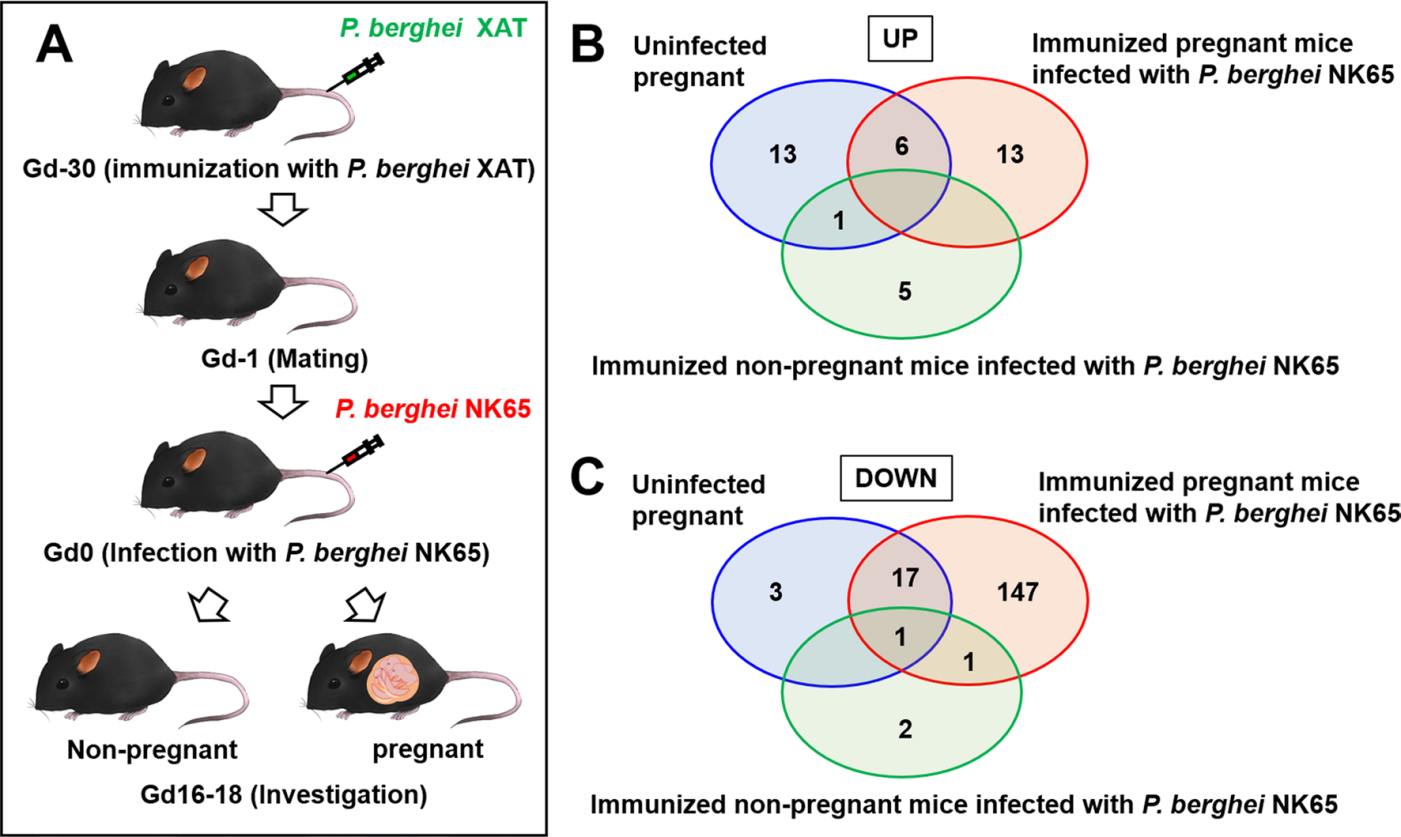
Proteomic analysis of the liver of immunized pregnant mice infected with malaria parasites. **A** Schematic representation of immunisation, mating, and infection. Female C57BL/6 (B6) mice were injected with 1 × 10^4^ erythrocytes infected with *Plasmodium berghei* XAT. Female mice were mated for 1 day and examined for the presence of a vaginal plug the following morning. Immunized mice with or without a vaginal plug were infected with 1 × 10^4^ erythrocytes infected with lethal *P. berghei* NK65. **B**, **C** Venn diagrams of protein levels in the livers of unimmunized pregnant mice without infection (naïve pregnant mice), immunized pregnant mice infected with *P. berghei* NK65, and non-pregnant mice infected with *P. berghei* NK65 that changed twofold (**B**) or 0.5-fold (**C**) compared with uninfected non-pregnant mice. Data are representative of three independent experiments

*Plasmodium berghei* XAT is an attenuated parasite and C57BL/6 mice cured of *P. berghei* XAT infection have acquired protective immunity that suppresses the severe pathology caused by the lethal malaria parasite *P. berghei* NK65 [24]. Mice immunized with the attenuated parasite with or without a vaginal plug were secondarily infected with the lethal malaria parasite *P. berghei* NK65, as described previously [12] (Fig. 1A).

To assess the proteome of the liver of IM pregnant C57BL/6 mice infected with *P. berghei* NK65, comparative proteomic analysis was performed using proteins extracted from the livers of unpregnant mice challenged with *P. berghei* NK65 and those of pregnant mice challenged with *P. berghei* NK65 (Fig. 1 and Tables 1, 2, and Additional file 1: Table S1). To investigate the effect of challenge infection on the proteomic alterations of liver during pregnancy, proteins extracted from the livers of unimmunized non-pregnant mice without infection (naïve virgin mice) and unimmunized pregnant mice without infection (naïve pregnant mice) were also analysed (Fig. 1 and Tables 1, 2, and Additional file 1: Table S1). A total of 1,758 proteins were detected, among which 1,159 proteins with three or more PSMs were analysed. Protein levels in the liver were normalized to actin, cytoplasmic 1 (accession: P60710) (Additional file 1: Table S1).

**Table 1:** Upregulated proteins in the liver of immunized pregnant mice infected with *P. berghei* NK65

Description	Abbreviation	Uninfected pregnant mouse	Immunized non-pregnant mouse infected with *P. berghei* NK65	Immunized pregnant mouse infected with *P. berghei* NK65	Accession
Perilipin-2 OS	Plin2	1.14	0.84	2.13	P43883
Pyruvate kinase PKM OS	Pkm	0.85	1.12	1.73	P52480*
Nicotinamide N-methyltransferase OS	Nnmt	0.52	0.57	1.13	O55239*
Interferon-inducible GTPase 1 OS	Iigp1	1.02	1.24	2.35	Q9QZ85*
Ferritin light chain 1 OS	Ftl1	0.33	1.61	0.79	P29391*
UMP-CMP kinase 2, mitochondrial OS	Cmpk2	1.01	1.02	2.45	Q3U5Q7*
Haptoglobin OS	Hp	1.49	0.82	4.09	Q61646*
Thymosin beta-10 OS	Tmsb10	0.63	3.58	1.82	Q6ZWY8*
Histone H2A type 1-H OS	Hist1h2ah	0.17	0.56	0.54	Q8CGP6*
Hemopexin OS	Hpx	0.85	0.88	2.67	Q91X72*
Signal transducer and activator of transcription 1 OS	Stat1	0.89	1.35	2.93	P42225*
Z-DNA-binding protein 1 OS	Zbp1	1.16	1.30	3.91	Q9QY24*
Coronin-1A OS	Coro1a	0.58	1.28	2.03	O89053*
Metallothionein-2 OS	Mt2	2.17	0.22	8.34	P02798*
Metallothionein-1 OS	Mt1	1.38	0.61	6.02	P02802*
Interferon-induced very large GTPase 1 OS	Gvin1	0.79	1.66	4.84	Q80SU7*
Interferon-induced protein with tetratricopeptide repeats 3 OS	Ifit3	0.54	1.41	9.25	Q64345*
Serum amyloid A-1 protein OS	Saa1	0.72	0.71	16.41	P05366*

Protein levels in the livers of immunized pregnant mice infected with *P. berghei* NK65 were compared with the levels in uninfected non-pregnant mice. Proteins with at least three peptide spectral matches and a fold change > 2.0 compared with unimmunized non-pregnant mice without infection (naïve virgin mice) are listed. Asterisks indicate proteins with significantly higher levels than in unimmunized non-pregnant mice without infection (naïve virgin mice) (> twofold change). Additional file 1: Table S1 shows all proteins detected in this study. Data are representative of three independent experiments

**Table 2:** Downregulated proteins in the liver of immunized pregnant mice infected with *P. berghei* NK65

Description	Abbreviation	Uninfected pregnant mouse	Immunized non-pregnant mouse infected with *P. berghei* NK65	Immunized pregnant mouse infected with *P. berghei* NK65	Accession
Major urinary protein 2 OS	Mup2	2.28	2.27	0.45	P11589†
Cytochrome P450 2C70 OS	Cyp2c70	1.99	0.97	0.47	Q91W64†
Atypical kinase ADCK3, mitochondrial OS	Adck3	0.62	0.97	0.15	Q60936†
Cytochrome P450 2A4 OS	Cyp2a4	1.77	0.29	0.44	P15392†
Pericentrin OS	Pcnt	0.84	0.67	0.23	P48725†
Fatty acid desaturase 2 OS	Fads2	1.53	0.82	0.45	Q9Z0R9†
Cytochrome P450 2C54 OS	Cyp2c54	0.79	1.11	0.26	Q6XVG2†
Guanidinoacetate N-methyltransferase OS	Gamt	1.41	1.05	0.47	O35969†
3-ketoacyl-CoA thiolase B, peroxisomal OS	Acaa1b	0.66	0.86	0.22	Q8VCH0†
Corticosteroid-binding globulin OS	Serpina6	8.48	0.21	2.84	Q06770†
Cytochrome P450 2C29 OS	Cyp2c29	1.15	1.24	0.39	Q64458†
D-dopachrome decarboxylase OS	Ddt	0.89	1.15	0.30	O35215†
Proteasome subunit beta type-5 OS	Psmb5	1.08	0.76	0.37	O55234†
6.8 kDa mitochondrial proteolipid OS	Mp68	0.88	0.82	0.31	P56379†
Apolipoprotein C-III OS	Apoc3	2.03	1.10	0.77	P33622†
Phenylalanine-4-hydroxylase OS	Pah	0.89	0.84	0.35	P16331†
Fatty acid synthase OS	Fasn	1.31	1.70	0.51	P19096†
Dimethylaniline monooxygenase [N-oxide-forming] 3 OS	Fmo3	2.31	1.41	0.92	P97501†

Protein levels in the livers of immunized pregnant mice infected with *P. berghei* NK65 were compared with levels in uninfected non-pregnant mice. Proteins with at least three peptide spectral matches and fold change < 0.5 compared with unimmunized non-pregnant mice without infection (naïve virgin mice) are listed. Daggers indicate proteins with significantly lower levels than in unimmunized non-pregnant mice without infection (naïve virgin mice) (< 0.4-fold change). Additional file 1: Table S1 shows all proteins detected in this study. Data are representative of three independent experiments

The levels of 13 proteins, including perilipin-2, which surrounds the lipid droplet [25], were significantly higher in IM pregnant mice infected with *P. berghei* NK65 than in naïve virgin mice (Fig. 1B and Table 1). These results are consistent with our previous findings showing that IM pregnant mice infected with *P. berghei* NK65 develop liver disease associated with microvesicular fatty infiltration [12]. Moreover, serum amyloid A-1 protein (Saa1) and interferon (IFN)-γ signalling pathway-related proteins, such as signal transducer and activator of transcription 1 (Stat1), IFN-inducible GTPase 1 (Iigp1), IFN-induced very large GTPase 1 (Gvin1), and IFN-induced protein with tetratricopeptide repeats 3 (Ifit3), were significantly higher in IM pregnant mice infected with *P. berghei* NK65 than in naïve virgin mice and immunized non-pregnant mice infected with *P. berghei* NK65 (Table 1). These results indicate that IFN-γ signalling contributes to the development of liver dysfunction during malaria in pregnancy.

By contrast, the levels of 147 proteins in IM pregnant mice infected with *P. berghei* NK65 were markedly decreased compared with naïve virgin mice (Fig. 1C and Additional file 1: Table S1). Of the 147 proteins, the levels of 18 proteins, including mitochondrial atypical kinase (ADCK3), peroxisomal 3-ketoacyl-CoA thiolase B (Acaa1b), and cytochrome P450 proteins, were significantly lower in IM pregnant mice infected with *P. berghei* NK65 than in naïve virgin mice (Table 2), indicating liver dysfunction.

### Effect of iNOS deficiency on microvesicular fatty infiltration in the liver

**Fig. 2:**
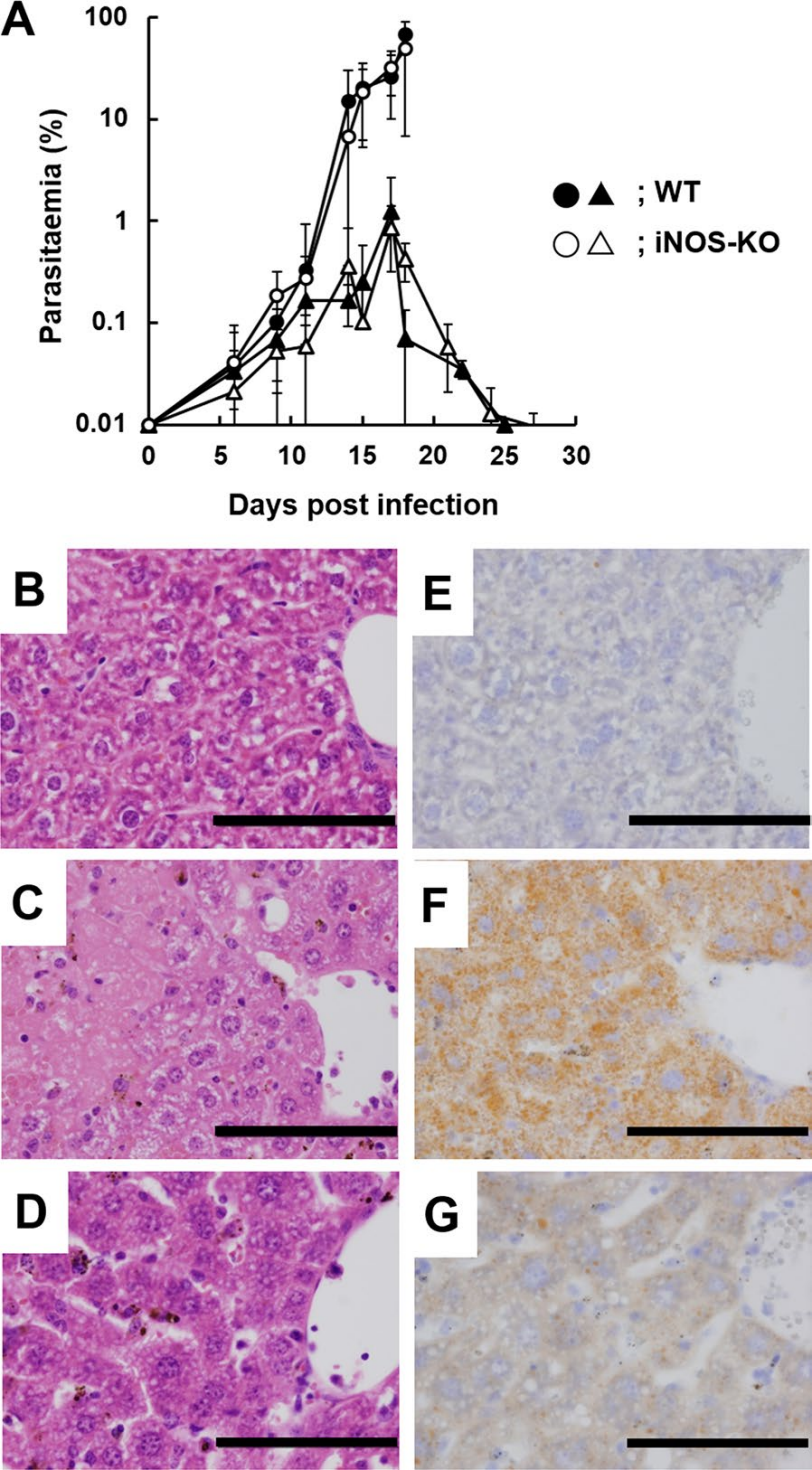
Nitric oxide produced by inducible nitric oxide synthase (iNOS) is associated with microvesicular fatty infiltration. Immunized female wild-type (WT) and iNOS-knockout (KO) mice were infected intravenously with 1 × 10^4^ erythrocytes infected with *P. berghei* NK65. **A** Course of parasitaemia. Closed and opened symbols indicate WT and iNOS-KO mice, respectively. Circles and triangles indicate pregnant and non-pregnant mice, respectively. Results are shown as the means ± standard deviation of three mice. The experiment was performed in triplicate with similar results. **B**–**G** Histological examination. Liver tissue staining with haematoxylin and eosin (**B**–**D**) and Sudan IV (**E**–**G**). Livers were obtained from mice on day 17 post-mating. **B**, **E** Immunized non-pregnant WT mice infected with *P. berghei* NK65. **C**, **F** Immunized pregnant WT mice infected with *P. berghei* NK65. **D**, **G** Immunized pregnant iNOS-KO mice infected with *P. berghei* NK65. Scale bar indicates 100 μm. Data are representative of six independent experiments

In previous study, the levels of IFN-γ and nitric oxide were increased in plasma from IM pregnant mice infected with *P. berghei* NK65 [12]. IFN-γ signalling triggers iNOS expression [26], and the free radical nitric oxide produced by iNOS has been implicated in fatty liver [27–29]. To investigate whether nitric oxide produced by iNOS is associated with microvesicular fatty infiltration, female wild-type (WT) and iNOS knockout (KO) mice immunized with the attenuated parasite *P. berghei* XAT, with or without a vaginal plug, were infected with *P. berghei* NK65. After infection with *P. berghei* NK65, immunized non-pregnant WT and iNOS-KO mice showed low levels of parasitaemia and were cured spontaneously (Fig. 2A). By contrast, IM pregnant iNOS-KO mice infected with *P. berghei* NK65 showed a rapid increase in parasitaemia (Fig. 2A). The course of parasitaemia in IM pregnant iNOS-KO mice infected with *P. berghei* NK65 was similar to that of IM pregnant WT mice infected with *P. berghei* NK65 (Fig. 2A). However, histological changes, such as microvesicular fatty infiltration, observed in the liver of IM pregnant WT mice infected with *P. berghei* NK65 were improved in IM pregnant iNOS-KO mice infected with *P. berghei* NK65 on day 17 post-mating (Fig. 2B–G).

**Fig. 3:**
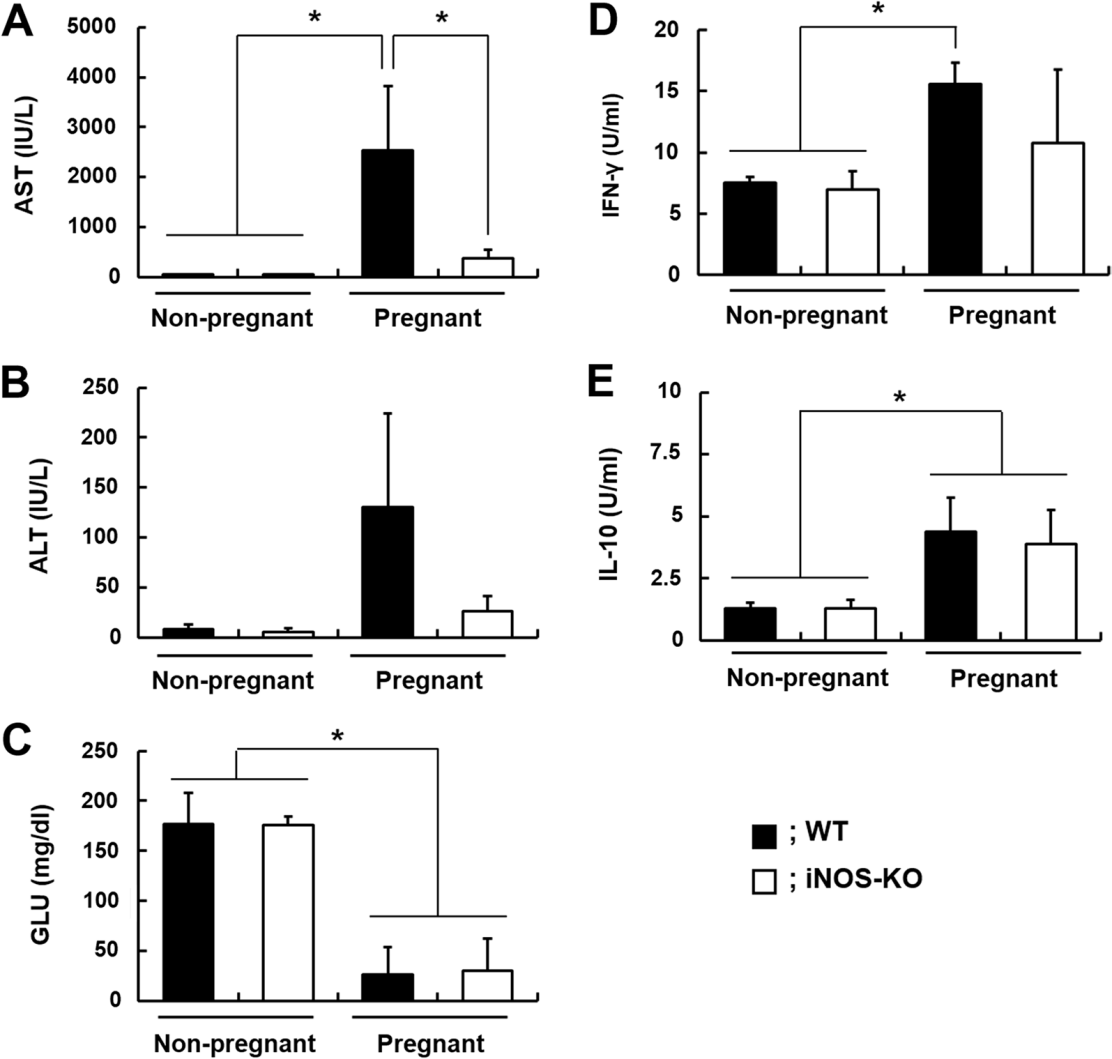
Biochemical examination of blood and cytokine levels. Samples of blood were obtained from immunized pregnant and non-pregnant mice infected with *P. berghei* NK65 on day 17 post-mating. Plasma aspartic aminotransferase (AST) (**A**), alanine aminotransferase (ALT) (**B**), glucose (GLU) (**C**), interferon (IFN)-γ (**D**), and interleukin (IL)-10 (**E**) levels are shown. Closed and opened symbols indicate wild-type (WT) and inducible nitric oxide synthase-knockout (iNOS-KO) mice, respectively. Non-pregnant indicate immunized non-pregnant WT and iNOS-KO mice infected with *P. berghei* NK65. Pregnant indicates immunized pregnant WT and iNOS-KO mice infected with *P. berghei* NK65. Asterisks indicate a statistically significant difference (*P* < 0.05 compared with immunized non-pregnant WT and iNOS-KO mice infected with *P. berghei* NK65; Tukey–Kramer and Dunnett test). Results are shown as the means ± standard deviation of three mice. Experiments were performed in triplicate with similar results

Increased levels of liver enzymes, such as aspartic aminotransferase, in IM pregnant WT mice infected with *P. berghei* NK65 on day 17 post-mating were significantly decreased in IM pregnant iNOS-KO mice infected with *P. berghei* NK65 (Fig. 3A, B). By contrast, levels of glucose in IM pregnant WT and iNOS-KO mice infected with *P. berghei* NK65 on day 17 post-mating were lower than those in immunized non-pregnant mice infected with *P. berghei* NK65 (Fig. 3C). These results suggest that nitric oxide produced by iNOS is associated with microvesicular fatty infiltration.

Unimmunized C57BL/6 mice infected with the lethal malaria parasite *P. berghei* NK65 exhibit increased parasitaemia in the early phase of infection and suffer from liver injury; all mice die within 2 weeks post-infection [30]. To investigate whether liver injury caused by iNOS-derived nitric oxide develops in unimmunized pregnant mice infected with *P. berghei* NK65, unimmunized pregnant WT and iNOS-KO mice on day 12 post-mating were infected with *P. berghei* NK65. Parasitaemia in unimmunized pregnant mice infected with *P. berghei* NK65 rapidly increased compared with unimmunized non-pregnant mice infected with *P. berghei* NK65 (Additional file 2: Fig. S1). This course of parasitaemia was not altered by iNOS deficiency (Additional file 2: Fig. S1). Histological analysis revealed that liver disease observed in infected non-pregnant and pregnant iNOS-KO mice was similar to that in infected WT mice (Additional file 2: Fig. S1). These results suggest that the impact of nitric oxide produced by iNOS is different in unimmunized and immunized mice.

### Effect of iNOS deficiency on the proteome in liver dysfunction

To investigate the effect of iNOS deficiency on the proteome of damaged livers of IM pregnant WT mice infected with *P. berghei* NK65, comparative proteomic analysis was performed (Tables 3, 4, and Additional file 3: Table S2). In the comparative proteomics analysis, 1,716 proteins were detected; of these, 1,154 proteins with three or more PSMs were analysed. Protein levels were normalized to actin, cytoplasmic 1 (accession: P60710) (Additional file 3: Table S2).

**Table 3:** Protein levels in the livers of immunized pregnant inducible nitric oxide synthase-knockout (iNOS-KO) mice infected with *P. berghei* NK65

Description	Abbreviation	iNOS-KO	Accession
Serum amyloid A-1 protein OS	Saa1	0.09	P05366*
Interferon-induced protein with tetratricopeptide repeats 3 OS	Ifit3	0.13	Q64345*
Metallothionein-2 OS	Mt2	0.27	P02798*
Ubiquitin-like protein ISG15 OS	Isg15	0.29	Q64339*
Ferritin light chain 1 OS	Ftl1	0.31	P29391*
T-cell-specific guanine nucleotide triphosphate-binding protein 1 OS	Tgtp1	0.32	Q62293*
Metallothionein-1 OS	Mt1	0.33	P02802*
Interferon-induced very large GTPase 1 OS	Gvin1	0.37	Q80SU7*
Perilipin-2 OS	Plin2	0.37	P43883*
Hemopexin OS	Hpx	0.41	Q91X72*
UMP-CMP kinase 2, mitochondrial OS	Cmpk2	0.41	Q3U5Q7*
Haptoglobin OS	Hp	0.42	Q61646*
Serine protease inhibitor A3K OS	Serpina3k	0.44	P07759*
E3 ubiquitin-protein ligase RNF213 OS	Rnf213	0.46	E9Q555*
Apolipoprotein A-IV OS	Apoa4	0.47	P06728*
Metalloreductase STEAP4 OS	Steap4	0.47	Q923B6*
Galectin-3-binding protein OS	Lgals3bp	0.49	Q07797*
Alpha-1-acid glycoprotein 8 OS	Orm8	0.50	P21352*
Coronin-1A OS	Coro1a	0.51	O89053
Pyruvate kinase PKM OS	Pkm	0.55	P52480
Fibrinogen gamma chain OS	Fgg	0.62	Q8VCM7
Signal transducer and activator of transcription 1 OS	Stat1	0.62	P42225
Fibrinogen beta chain OS	Fgb	0.63	Q8K0E8
Neutrophilic granule protein OS	Ngp	0.63	O08692
Cytochrome P450 4A14 OS	Cyp4a14	0.63	O35728
Fructose-bisphosphate aldolase A OS	Aldoa	0.64	P05064
Nicotinamide N-methyltransferase OS	Nnmt	0.66	O55239
Thymosin beta-4 OS	Tmsb4x	0.67	P20065
Glycogen [starch] synthase, liver OS	Gys2	0.68	Q8VCB3
Dipeptidyl peptidase 1 OS	Ctsc	0.70	P97821
Intercellular adhesion molecule 1 OS	Icam1	0.71	P13597
Interferon-inducible GTPase 1 OS	Iigp1	0.71	Q9QZ85
Histone H4 OS	Hist1h4a	0.73	P62806

Protein levels in the livers of immunized pregnant iNOS-KO mice infected with *P. berghei* NK65 compared with immunized pregnant wild-type (WT) mice infected with *P. berghei* NK65. Proteins with at least three peptide spectral matches and fold change < 0.735 compared with immunized pregnant WT mice infected with *P. berghei* NK65 are listed. Asterisks indicate proteins with significantly lower levels than in immunized pregnant WT mice infected with *P. berghei* NK65 (< 0.5-fold change). Additional file 3: Table S2 shows all proteins detected in this study. Data are representative of three independent experiments

**Table 4:** Upregulated proteins in the livers of immunized pregnant inducible nitric oxide synthase-knockout (iNOS-KO) mice infected with *P. berghei* NK65

Description	Abbreviation	iNOS-KO	Accession
Cytochrome P450 1A2 OS	Cyp1a2	2.00	P00186
Glutathione S-transferase A2 OS	Gsta2	2.01	P10648
Sulfide:quinone oxidoreductase, mitochondrial OS	Sqrdl	2.03	Q9R112
Carbonic anhydrase 3 OS	Ca3	2.08	P16015
Serum paraoxonase/arylesterase 1 OS	Pon1	2.09	P52430
L-gulonolactone oxidase OS	Gulo	2.11	P58710
Cytochrome P450 2C29 OS	Cyp2c29	2.13	Q64458
Guanidinoacetate N-methyltransferase OS	Gamt	2.13	O35969
Dimethylaniline monooxygenase [N-oxide-forming] 3 OS	Fmo3	2.16	P97501
Antithrombin-III OS	Serpinc1	2.22	P32261
Methyltransferase-like protein 7B OS	Mettl7b	2.24	Q9DD20
Cytochrome P450 2A4 OS	Cyp2a4	2.36	P15392
Pericentrin OS	Pcnt	2.66	P48725
Cytochrome P450 2C37 OS	Cyp2c37	2.66	P56654
Corticosteroid-binding globulin OS	Serpina6	3.19	Q06770
Cytochrome P450 2C54 OS	Cyp2c54	3.91	Q6XVG2
Cytochrome P450 2C70 OS	Cyp2c70	4.20	Q91W64

Protein levels in the livers of immunized pregnant iNOS-KO mice infected with *P. berghei* NK65 compared with immunized pregnant WT mice infected with *P. berghei* NK65. Proteins with at least three peptide spectral matches and fold change > 2.0 compared with immunized pregnant WT mice infected with *P. berghei* NK65 are listed. Additional file 3: Table S2 shows all proteins detected in this study. Data are representative of three independent experiments

The levels of 18 proteins, including perilipin-2, were markedly decreased in IM pregnant iNOS-KO mice infected with *P. berghei* NK65 compared with IM pregnant WT mice infected with *P. berghei* NK65 (Table 3). These results support our results that histological changes were improved in IM pregnant iNOS-KO mice infected with *P. berghei* NK65 (Fig. 2B–G). Moreover, Saa1 and IFN-γ signalling pathway-related proteins, such as Gvin1 and Ifit3, were significantly lower in IM pregnant iNOS-KO mice infected with *P. berghei* NK65 than in IM pregnant WT mice infected with *P. berghei* NK65 (Table 3). However, Stat1 and Iigp1 levels in IM pregnant iNOS-KO mice infected with *P. berghei* NK65 were comparable to those in IM pregnant WT mice infected with *P. berghei* NK65, suggesting that IFN-γ signalling was activated in IM pregnant iNOS-KO mice infected with *P. berghei* NK65. In addition, the levels of IFN-γ and interleukin-10 in plasma were similar between IM pregnant WT and iNOS-KO mice infected with *P. berghei* NK65 (Fig. 3D, E).

By contrast, decreased levels of proteins, such as cytochrome P450 proteins (Table 2), in IM pregnant WT mice infected with *P. berghei* NK65 were improved in IM pregnant iNOS-KO mice infected with *P. berghei* NK65 (Table 4). These results indicate that nitric oxide produced by iNOS is involved in the development of liver dysfunction during malaria in pregnancy.

### Effect of iNOS deficiency on pregnancy outcomes

**Fig. 4:**
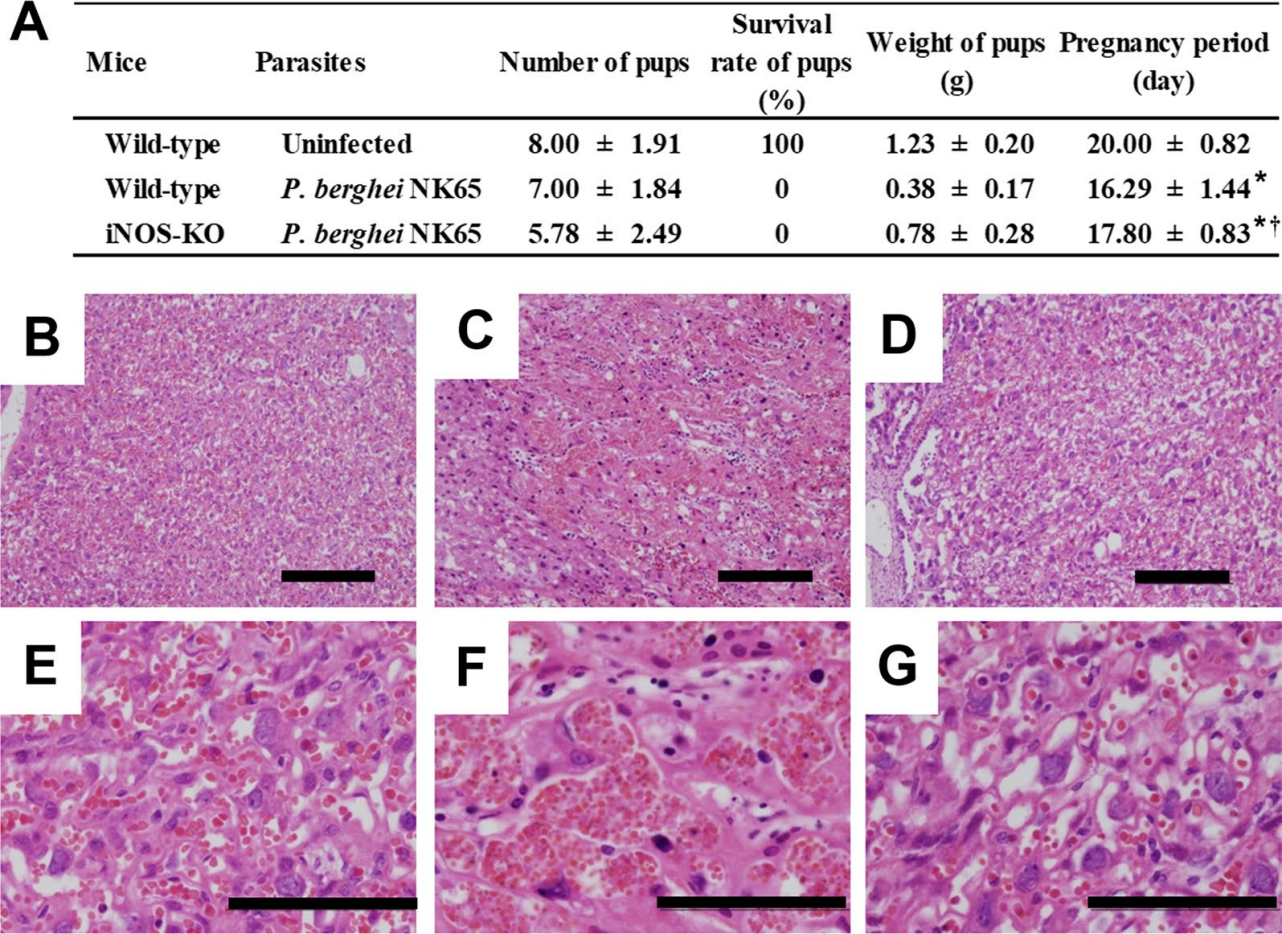
Inducible nitric oxide synthase (iNOS) may be involved in adverse pregnancy outcomes during malaria. **A** Pregnancy outcomes in uninfected pregnant wild-type (WT) mice and immunized pregnant WT and iNOS-knockout (KO) mice infected with *P. berghei* NK65. Number, survival rate, and weight of fetuses were measured at delivery. Experiments using three mice were performed in triplicate with similar results. Asterisks indicate a significant difference compared with the pregnancy period of uninfected pregnant WT mice (*P* < 0.05, Tukey–Kramer and Dunnett test). Dagger indicates a significant difference compared with the pregnancy period of immunized pregnant WT mice infected with *P. berghei* NK65. **B**–**G** Representative haematoxylin and eosin-stained placental sections are shown. Placentas were obtained from uninfected pregnant WT mice and immunized pregnant WT and iNOS-KO mice infected with *P. berghei* NK65 on day 17 post-mating. **B**–**D** Low magnification images of placentas. Scale bars indicate 200 μm. **E**–**G** Higher magnification of the labyrinth region. Scale bars indicate 100 μm. **B**, **E** Uninfected pregnant WT mice. **C**, **F** Immunized pregnant WT mice infected with *P. berghei* NK65. **D**, **G** Immunized pregnant iNOS-KO mice infected with *P. berghei* NK65. Data are representative of six independent experiments

Stillbirth was observed in IM pregnant WT and iNOS-KO mice infected with *P. berghei* NK65. However, the pregnancy period of IM pregnant iNOS-KO mice infected with *P. berghei* NK65 was longer than that of IM pregnant WT mice infected with *P. berghei* NK65 (Fig. 4A). Accumulation of infected erythrocytes and a decreasing number of vascular branches were observed in the placentas of IM pregnant WT mice infected with *P. berghei* NK65 on days 16–18 post-mating, but not in IM pregnant iNOS-KO mice infected with *P. berghei* NK65 (Fig. 4B–G). These results suggest that nitric oxide produced by iNOS is involved in the enhancement of placental inflammation during malaria in pregnancy.

## Discussion

Pregnant women infected with malaria parasites develop liver disease [8–10], but its pathogenesis remains unclear. Here, comparative proteomic analysis of the liver in a mouse model of malaria in pregnancy was performed. This analysis revealed that the levels of IFN-γ signalling pathway-related proteins were increased in the livers of IM pregnant mice infected with *P. berghei* NK65. These results suggest that the inflammation induced by IFN-γ signalling is involved in the development of liver disease during malaria in pregnancy.

In this study, the levels of Saa1 were increased in the liver of IM pregnant mice infected with *P. berghei* NK65. Increased levels of Saa have also been observed in plasma samples from HELLP patients [31]. Meanwhile, the levels of cytochrome P450 proteins, such as Cyp2a4, Cyp2c29, and Cyp2c70, were decreased in the liver during malaria in pregnancy. The levels of cytochrome P450 proteins are altered in patients with non-alcoholic fatty liver disease [32] and a corresponding animal model [33, 34]. These findings suggest that inflammation or infection affect levels of cytochrome P450 proteins in the liver.

iNOS-derived nitric oxide is thought to inhibit fatty acid oxidation. In fact, inhibition of β-oxidation is involved in the pathology of acetaminophen-induced hepatotoxicity [35]. However, iNOS-KO mice are resistant to hepatotoxicity caused by acetaminophen [36]. Decreased levels of mitochondrial and peroxisomal proteins were observed in the liver during malaria in pregnancy. While it is unclear whether iNOS-derived nitric oxide directly inhibits mitochondrial and peroxisomal proteins, results showing improvement of these protein levels by iNOS deficiency suggest that iNOS-derived nitric oxide may be involved in mitochondrial or peroxisomal dysfunction in the liver during malaria in pregnancy.

*Plasmodium berghei* XAT is an attenuated parasite and mice infected with *P. berghei* XAT show low levels of parasitaemia and become cured spontaneously [37]. The course of parasitaemia and production of IFN-γ are not altered by iNOS deficiency [38]. In this study, when *P. berghei* XAT-immunized mice were infected with the lethal malaria parasite *P. berghei* NK65, their course of parasitaemia was not altered by iNOS deficiency. These findings indicate that acquisition of protective immunity by infection with malaria parasites is iNOS independent.

Liver disease associated with microvesicular fatty infiltration during malaria in pregnancy was improved by iNOS deficiency. It has been suggested that stress by alcohol or a high-cholesterol diet induce high levels of iNOS mRNA expression and cause fatty liver disease. These fatty liver diseases are improved by iNOS deficiency [27, 28], suggesting that iNOS plays a pivotal role in the development of liver disease. These results suggest that iNOS induced by inflammation or infection is an important mediator in liver disease during malaria in pregnancy, in addition to alcohol- or high-cholesterol diet-induced fatty liver disease.

In contrast to findings that nitric oxide produced by iNOS in inflammation is detrimental to organs, nitric oxide supplied by l-arginine and the inhalation of nitric oxide were shown to have a protective/beneficial effect during severe malaria [39–42]. There are three isozymes that produce nitric oxide: nNOS (neuronal NOS, NOS1), iNOS (inducible NOS, NOS2), and eNOS (endothelial NOS, NOS3). eNOS plays an important role in maintaining endothelial homeostasis, such as angiogenesis and platelet production [43]. iNOS generates a larger amount of nitric oxide than eNOS or nNOS and is involved in cytotoxic functions [44]. l-Arginine supplementation and inhaled nitric oxide regulate angiopoietin levels and von Willebrand factor release [39–42]. These findings suggest that l-arginine and the inhalation of nitric oxide supply an appropriate amount of nitric oxide and play an auxiliary role alongside eNOS.

In this study, levels of the anti-inflammatory cytokine IL-10 in plasma were elevated in pregnant mice. During pregnancy, IL-10-producing regulatory T cells expand, conferring immune tolerance [45]. In IM pregnant mice infected with *P. berghei* NK65, this would suppress the acquired immunity against malaria parasites, thus increasing parasitaemia. The development of liver disease in IM pregnant WT mice infected with *P. berghei* NK65 suggested that the development of liver disease during malaria in pregnancy was not suppressed by IL-10. A previous study showed that liver damage during *P. berghei* NK65 induced infection was mediated by IL-27 in an IL-10 independent manner [46]. These findings point to a role for IL-27 in suppressing the development of liver disease during malaria in pregnancy. The elevated serum level of IL-27 in women with severe preeclampsia suggests IL-27 as a useful biomarker for disease severity in preeclampsia [47].

In IM iNOS-KO mice infected with *P. berghei* NK65, all infants were stillborn. These results suggest that nitric oxide produced by iNOS is not essential for foetal survival. However, pregnancy was longer in IM iNOS-KO mice infected with *P. berghei* NK65 than in IM WT mice infected with *P. berghei* NK65. Placental pathology observed in IM pregnant WT mice infected with *P. berghei* NK65 was not observed in IM pregnant iNOS-KO mice infected with *P. berghei* NK65. These results suggest that nitric oxide produced by iNOS may be involved in the enhancement of placental inflammation. Oxidative stress in placental mitochondria and peroxisomes may play a role in foetal fatty acid oxidation disorders during pregnancy [48, 49]. Based on these findings, placental pathology may be associated with maternal liver diseases during malaria in pregnancy.

In contrast to IM pregnant mice infected with *P. berghei* NK65, liver disease in unimmunized pregnant mice infected with *P. berghei* NK65 was not improved by iNOS deficiency. CD8^+^ T cells have been shown to play a pivotal role in the development of liver disease in unimmunized mice infected with *P. berghei* NK65 [30]. From these findings, the contribution of iNOS is considered to be much less than that of CD8^+^ T cells in the development of liver disease in unimmunized mice infected with *P. berghei* NK65. By contrast, the immune response induced in IM pregnant mice infected with *P. berghei* NK65 may differ from that in unimmunized pregnant mice infected with *P. berghei* NK65 because immunized mice have acquired protective immunity against malaria parasites, suggesting that iNOS may play a role in maternal liver diseases in pregnant women living in malaria-endemic areas.

In this study, alteration of the liver proteome during malaria in pregnancy was detected. Because differentiating HELLP-like syndrome during malaria in pregnancy can be challenging, the identification of biomarkers is necessary. The decreased levels of mitochondrial and peroxisomal proteins observed in this study are potential biomarkers of liver disease during malaria in pregnancy. Furthermore, a causative role of iNOS in liver disease associated with microvesicular fatty infiltration during malaria in pregnancy was demonstrated in this study. iNOS is known to be associated with metabolic diseases. In vivo models of liver disease during malaria in pregnancy will further our understanding of the role of the iNOS-mediated metabolic response and the pathogenesis of high-risk liver diseases of pregnancy, such as acute fatty liver.

## Conclusions

iNOS plays a causative role in the development of liver disease during malaria in pregnancy.

## Supplementary Information

**Additional file 1: Table S1.** Comparative proteomic analysis of livers of immunized pregnant mice infected with *P. berghei* NK65. Livers were obtained from unimmunized pregnant mice without infection (naïve pregnant mice) and unimmunized non-pregnant mice without infection (naïve virgin mice), and immunized pregnant and non-pregnant mice infected with *P. berghei* NK65 on day 17 post-mating. Proteins showing one or two peptide spectral matches were excluded. Protein levels were normalized to actin, cytoplasmic 1 (accession: P60710).

**Additional file 2: Figure S1.** Nitric oxide produced by inducible nitric oxide synthase (iNOS) is not involved in the development of liver injury during primary infection with lethal malaria parasites. Unimmunized wild-type (WT) and iNOS-knockout (KO) female mice were placed with male WT mice for 1 day. Mice on day 12 post-mating were infected with 1 × 10^4^ erythrocytes infected with *Plasmodium berghei* NK65. (A) Course of parasitaemia. Closed and opened symbols indicate WT and iNOS-KO mice, respectively. Circles and triangles indicate pregnant and non-pregnant mice, respectively. Results are shown as the means ± standard deviation of three mice. (B–E) Liver tissue staining with haematoxylin and eosin. Livers were obtained from mice on day 7 post-infection. (B) Unimmunized non-pregnant WT mice infected with *P. berghei* NK65. (C) Unimmunized pregnant WT mice infected with *P. berghei* NK65. (D) Unimmunized non-pregnant iNOS-KO mice infected with *P. berghei* NK65. (E) Unimmunized pregnant iNOS-KO mice infected with *P. berghei* NK65. Scale bar indicates 100 μm. Data are representative of three independent experiments.

**Additional file 3: Table S2.** Comparative proteomic analysis of livers of immunized pregnant inducible nitric oxide synthase-knockout (iNOS-KO) mice infected with *P. berghei* NK65. Livers were obtained from immunized pregnant mice infected with *P. berghei* NK65 on day 17 post-mating. Proteins showing one or two peptide spectral matches were excluded. Protein levels were normalized to actin, cytoplasmic 1 (accession: P60710).

## Data Availability

All data generated or analysed during this study are included in this published article and its additional files.

## Abbreviations

AFLP: Acute fatty liver of pregnancy
ALT: Alanine aminotransferase
AST: Aspartic aminotransferase
HELLP: Hemolytic anemia; elevated liver enzymes; low platelet count
iNOS: Inducible nitric oxide synthase
LCHAD: Long-chain 3-hydroxyacyl CoA dehydrogenase
NAFLD: Non-alcoholic fatty liver disease
PSMs: Peptide spectral matches
