# Supplementary material for: The association between acute fatty liver disease and nitric oxide during malaria in pregnancy
Source: Malar J. 2021 Dec 14;20:462. doi: 10.1186/s12936-021-03999-2 (PMC8670279; doi:10.1186/s12936-021-03999-2)
Supplement: Supplementary file 2 — Additional file 2: Figure S1. Nitric oxide produced by inducible nitric oxide synthase (iNOS) is not involved in the development of liver injury during primary infection with lethal malaria parasites. Unimmunized wild-type (WT) and iNOS-knockout (KO) female mice were placed with male WT mice for 1 day. Mice on day 12 post-mating were infected with 1 × 10^4^ erythrocytes infected with Plasmodium berghei NK65. (A) Course of parasitaemia. Closed and opened symbols indicate WT and iNOS-KO mice, respectively. Circles and triangles indicate pregnant and non-pregnant mice, respectively. Results are shown as the means ± standard deviation of three mice. (B–E) Liver tissue staining with haematoxylin and eosin. Livers were obtained from mice on day 7 post-infection. (B) Unimmunized non-pregnant WT mice infected with P. berghei NK65. (C) Unimmunized pregnant WT mice infected with P. berghei NK65. (D) Unimmunized non-pregnant iNOS-KO mice infected with P. berghei NK65. (E) Unimmunized pregnant iNOS-KO mice infected with P. berghei NK65. Scale bar indicates 100 μm. Data are representative of three independent experiments. [file 12936_2021_3999_MOESM2_ESM.pdf]

The association between acute fatty liver disease and nitric oxide during malaria in pregnancy
Short title: Liver disease during malaria in pregnancy

Mamoru Niikura, Toshiyuki Fukutomi, Shoichiro Mineo, Jiro Mitobe, Fumie Kobayashi

Supplemental Figure

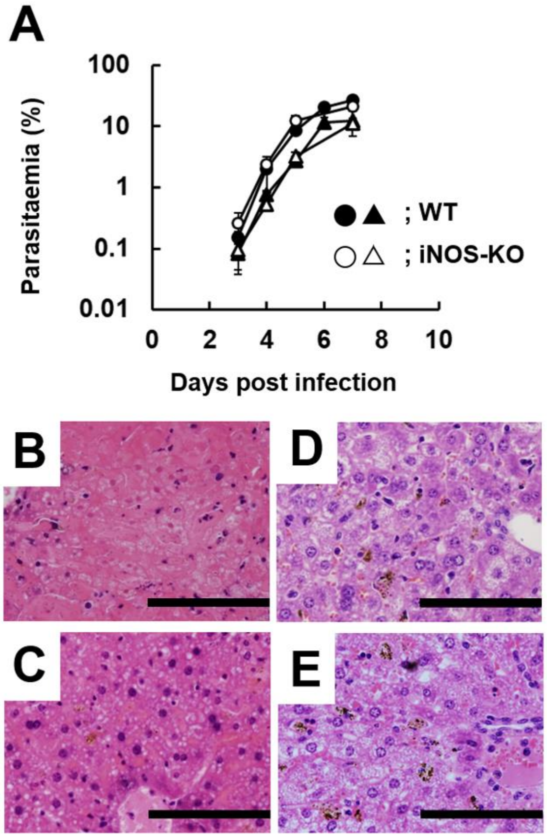

Figure S1. Nitric oxide produced by inducible nitric oxide synthase (iNOS) is not involved in the development of liver injury during primary infection with lethal malaria parasites.
Unimmunized wild-type (WT) and iNOS-knockout (KO) female mice were placed with male WT mice for 1 day. Mice on day 12 post-mating were infected with 1×10^4 erythrocytes infected with *Plasmodium berghei* NK65. (A) Course of parasitaemia. Closed and opened symbols indicate WT and iNOS-KO mice, respectively. Circles and triangles indicate pregnant and non-pregnant mice, respectively. Results are shown as the means ± standard deviation of three mice. (B–E) Liver tissue staining with haematoxylin and eosin. Livers were obtained from mice on day 7 post-infection. (B) Unimmunized non-pregnant WT mice infected with *P. berghei* NK65. (C) Unimmunized pregnant WT mice infected with *P. berghei* NK65. (D) Unimmunized non-pregnant iNOS-KO mice infected with *P. berghei* NK65. (E) Unimmunized pregnant iNOS-KO mice infected with *P. berghei* NK65. Scale bar indicates 100 µm. Data are representative of three independent experiments.
